# Microbiome Alterations in Alcohol Use Disorder and Alcoholic Liver Disease

**DOI:** 10.3390/ijms24032461

**Published:** 2023-01-27

**Authors:** Kamil Litwinowicz, Andrzej Gamian

**Affiliations:** 1 Department of Biochemistry and Immunochemistry, Wroclaw Medical University, 50-368 Wroclaw, Poland; 2 Hirszfeld Institute of Immunology and Experimental Therapy, Polish Academy of Sciences, 53-114 Wrocław, Poland

**Keywords:** alcohol, gut microbiota, microbiome, liver disease

## Abstract

Microbiome alterations are emerging as one of the most important factors that influence the course of alcohol use disorder (AUD). Recent advances in bioinformatics enable more robust and accurate characterization of changes in the composition of the microbiome. In this study, our objective was to provide the most comprehensive and up-to-date evaluation of microbiome alterations associated with AUD and alcoholic liver disease (ALD). To achieve it, we have applied consistent, state of art bioinformatic workflow to raw reads from multiple 16S rRNA sequencing datasets. The study population consisted of 122 patients with AUD, 75 with ALD, 54 with non-alcoholic liver diseases, and 260 healthy controls. We have found several microbiome alterations that were consistent across multiple datasets. The most consistent changes included a significantly lower abundance of multiple butyrate-producing families, including *Ruminococcaceae*, *Lachnospiraceae*, and *Oscillospiraceae* in AUD compared to HC and further reduction of these families in ALD compared with AUD. Other important results include an increase in endotoxin-producing *Proteobacteria* in AUD, with the ALD group having the largest increase. All of these alterations can potentially contribute to increased intestinal permeability and inflammation associated with AUD and ALD.

## 1. Introduction

Alcohol use disorder (AUD) is one of the most significant contributors to the global burden of mortality and disease [1]. It is a risk factor for more than 200 diseases, of which more than 40 are fully attributable to alcohol [2]. Of these, alcoholic liver disease (ALD) is the largest contributor to the health harm caused by AUD [1]. ALD is responsible for almost 50% of all deaths attributed to liver diseases in adults [1]. Considering the significant increase in the prevalence of AUD [3] and, in the best case, the moderate effectiveness of currently approved therapies [4], new therapeutic approaches are urgently needed.

ALD is divided into several stages, ranging from relatively benign and reversible hepatic steatosis to severe and irreversible cirrhosis. Hepatic steatosis will affect 90% of heavy drinkers, however, only 10–35% will progress to alcoholic steatohepatitis (ASH) and 8–20% to cirrhosis [5]. Identification of individuals at increased risk of progression to more advanced stages of ALD could potentially help the development of novel and more personalized approaches for the treatment and prevention of ALD by, for example, finding patients who could potentially benefit from early fecal microbiota transplantation from a donor with desired microbiome composition. Although significant advances have been made in recent years, the pathophysiology of ALD development and progression remains largely unknown. Multiple, partially overlapping mechanisms have been proposed as potential causes of liver injury, including advanced glycation end-products [6], oxidative stress [7], and genetic factors [8]. A growing body of evidence shows that microbiome alterations are another factor that influences the development and course of ALD [9].

Significant technological advances have reduced the cost of sequencing faster than predicted by Moore’s law [10]. This has enabled the development of cultivation-independent methods for studying the human microbiome. Of these methods, due to their low cost and high accuracy, 16S rRNA sequencing remains the most widely used. Rapid progress in sequencing technologies is accompanied by significant advances in bioinformatic methods associated with the study of the microbiome. Advances include novel denoising methods, enabling better removal of spurious sequences caused by PCR errors [11], more accurate taxonomic classification methods [12], and more robust methods for differential abundance (DA) testing [13]. Although often overlooked, the impact of the choice of correct bioinformatic methods for microbiome analysis is tremendous; in some cases, frequently used tools for differential abundance testing have been shown to have a false discovery rate (FDR) as high as 70%, meaning that up to 70% of bacteria discovered as significant could be wrong [13]. Analysis of 14 differential abundance testing methods on 38 datasets has shown that the proportion of bacteria discovered as significant strongly depends on the used method; for example, the use of the Wilcoxon test on data normalized as centered log ratio (CLR) resulted in the detection of up to 90% of bacteria as significantly different between studied groups; when using ANCOM-II on the same datasets, on average only 0.8% of bacteria were detected as significant. The authors concluded that ANCOM-II produced the most consistent and conservative results of all the methods studied [14]. The recently developed novel method for differential abundance testing called ANCOM-BC provides further improvement over ANCOM-II, with an additional reduction in FDR while maintaining adequate power [13].

Studies examining the microbiome in AUD used different, and in some cases, suboptimal bioinformatic tools for reads preparation and differential abundance testing, including statistical tests which do not account for the compositional nature of the microbial abundance data, lack of adjustment for multiple testing, and tools with high FDR such as LEfSe. As discussed above, these inconsistencies could potentially influence the obtained results. For this reason, to provide a more accurate and consistent assessment of the microbiome, we have applied a standard workflow consisting of state-of-the-art bioinformatics methods characterized by the best performance in benchmarking studies to datasets that examine the microbiome composition in AUD and ALD. In addition, the inclusion of multiple datasets has enabled us to use dataset identifiers as confounding variable, which reduced variability due to non-bioinformatic technical differences, such as choice of primers and DNA extraction methods.

## 2. Results

### 2.1. Diversity Analysis and Variance Contribution

**Figure 1:**
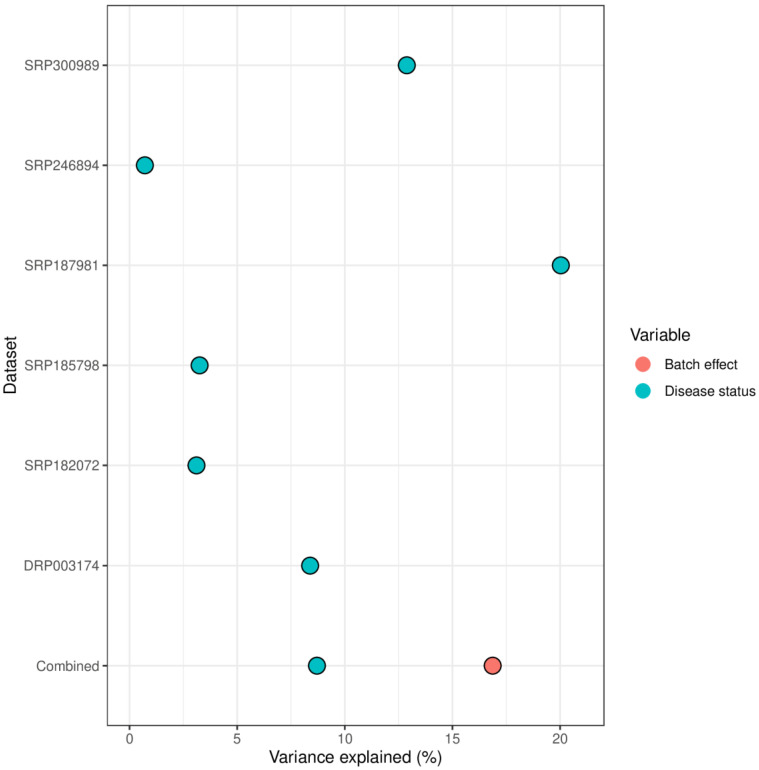
Microbiome variance explained by batch effect (i.e., dataset ID) and disease status (alcohol use disorder vs healthy control).

The variance contribution of the disease status differed significantly between the datasets and ranged from less than 1% to 20% (Figure 1). Most studies reported variance contribution of disease status smaller than 10%. In the combined dataset approach, the variance contribution of the batch effect was approximately two times larger than that of disease status.

**Figure 2:**
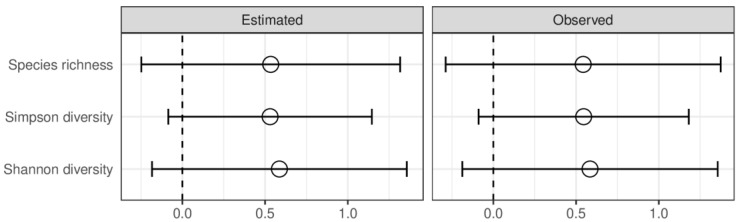
Observed and estimated alpha diversity for comparison between patients with alcohol use disorder and healthy controls denoted as Agresti’s generalized odds ratio and summarized with random effects model; values greater than 0 indicate greater richness in healthy controls. An open dot denotes statistically insignificant results. Error bars indicate 95% confidence intervals.

**Figure 3:**
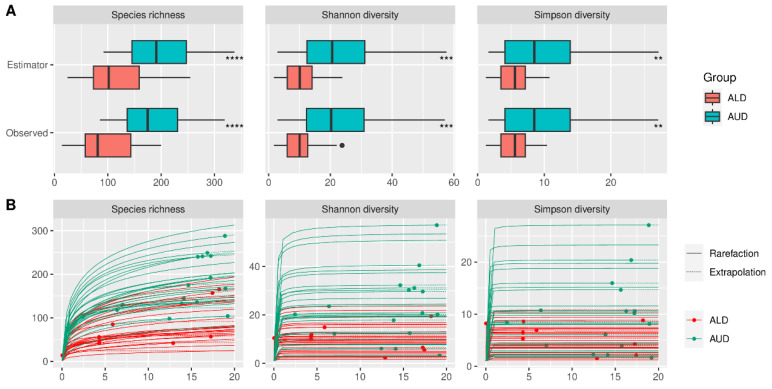
(**A**) Observed and estimated alpha diversity for comparison between patients with alcoholic liver disease and alcohol use disorder (without liver disease). (**B**) Rarefication–extrapolation curves for alpha-diversity measures. Each curve represents the alpha diversity of a single sample. Dots represent observed read depths, which were extrapolated to a common read depth of 20,000. Curves without dots indicate samples that had the read depth already above 20,000 and thus were rarefied to that level; ALD—alcoholic liver disease, AUD—alcohol use disorder, **—*p* < = 0.01, ***—*p* < = 0.001, ****—*p* < = 0.0001.

**Figure 4:**
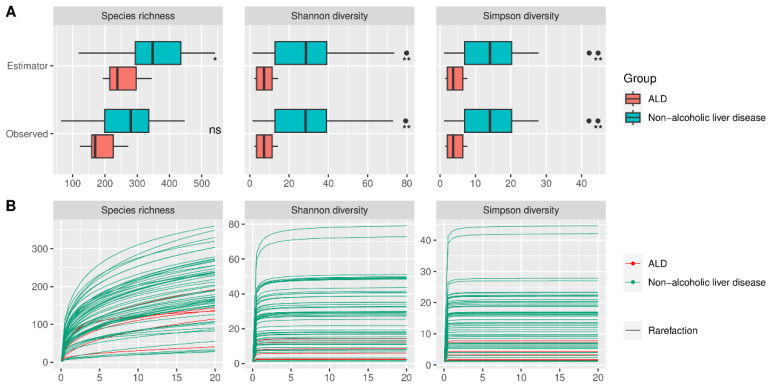
(**A**) Observed and estimated alpha diversity for comparison between patients with alcoholic liver disease and patients with other, non-alcoholic etiologies of liver disease. Black dots denote outliers. (**B**) Rarefication–extrapolation curves for alpha-diversity measures. Each curve represents the alpha diversity of a single sample rarefied to a common read depth of 20,000; ALD—alcoholic liver disease, *—*p* < = 0.05, **—*p* < = 0.01, ns—non-significant.

Alpha diversity did not differ significantly between HC and AUD, both for estimated and observed measures (Figure 2). The rarefication–extrapolation curves for these comparisons are provided in Supplementary Figure S2. Patients with ALD had a lower alpha diversity for all three measures compared with AUD for both estimated and observed measures (Figure 3). The non-alcoholic liver diseases group had higher alpha diversity than the ALD group; however, the species richness differed significantly only for estimated values (Figure 4).

**Figure 5:**
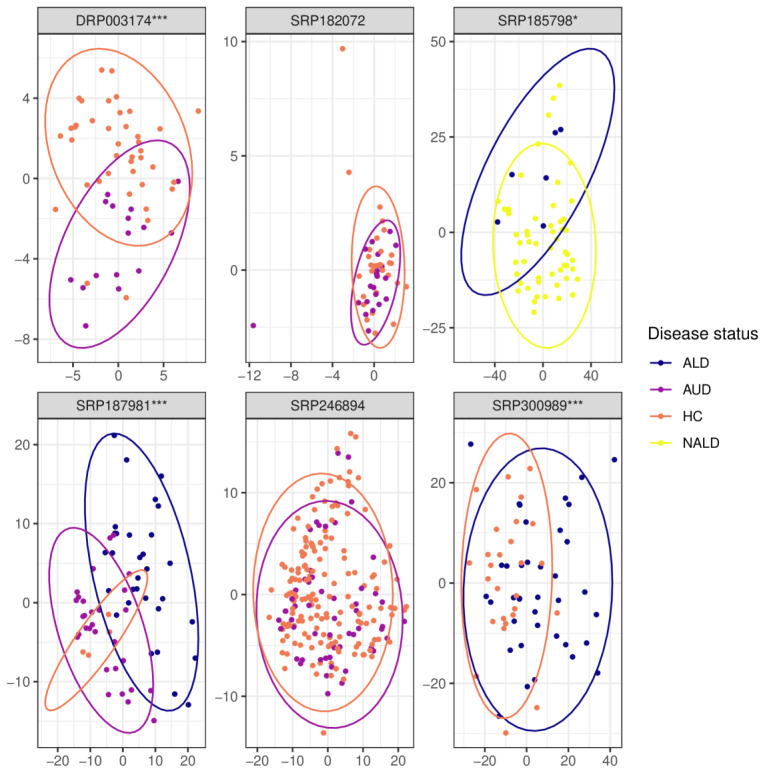
Principal coordinate analysis (PCoA) plots of beta-diversity measured with Euclidean distance after normalization using PhilR. The label above each graph indicates the SRA identifier for the corresponding dataset; ALD—alcoholic liver disease, AUD—alcohol use disorder, HC—healthy controls, NALD—other, non-alcoholic liver disease, *—*p* < = 0.05, ***—*p* < = 0.001.

In three out of five data sets (DRP003174, SRP187981, and SRP300989), beta diversity was significantly different for the comparison of HC versus AUD (Figure 5). Beta-diversity differed significantly also for the ALD vs. nonalcoholic liver disease and AUD vs ALD comparison (both examined by one dataset each, SRP185798 and SRP187981, respectively).

### 2.2. Alcohol Consumption Causes Unfavorable Changes in the Fecal Microbiome

**Figure 6:**
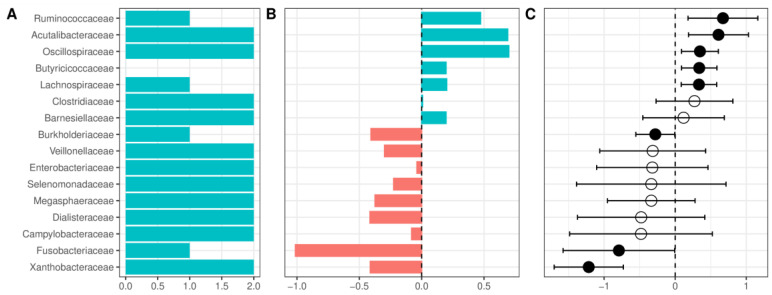
Changes in the relative abundance between patients with alcohol use disorder and healthy controls at the family level. (**A**) Number of datasets in which the given family was detected as significant. (**B**) Change in the relative abundance of a given family in the combined dataset reported as log-ratio. Blue denotes bacteria more abundant in healthy controls, and red more abundant in alcohol use disorder. (**C**) Change in the relative abundance of a given family after summarizing results from individual datasets using the random-effects model, expressed as a mean difference in centered log ratios. Values larger than zero denote greater abundance in healthy controls. A full dot denotes a statistically significant effect. Error bars indicate 95% confidence intervals. Only bacteria detected as significant in at least two datasets or significant in the random-effects analysis are presented.

The complete results for all comparisons at each of the studied taxonomic levels are presented in Figure 6 and Supplementary Figures S3–S8.

### 2.3. Alcohol Use Disorder versus Healthy Controls

At the phylum level, patients with AUD had a lower relative abundance of *Firmicutes*, *Cyanobacteria*, and a *Verrucomicrobiota*, and a higher abundance of *Proteobacteria* and *Fusobacteria* (Supplementary Figure S3). The decrease in the relative abundance of *Firmicutes* was accompanied by a nonsignificant decrease of the *Firmicutes*/*Bacteroidetes* ratio (F/B ratio, a frequently used marker of gut health) in most of the datasets. This result remained nonsignificant after pooling with a random-effects model (Supplementary Figure S9). At the class level, patients with AUD were enriched in *Fusobateria*, *Gammaproteobacteria*, and *Negativicutes*, whereas HC had a higher abundance of *Clostridia*, *Verrucomicrobiae*, and *Alphaproteobacteria* (however, several classes had very wide confidence interval crossing 0, Supplementary Figure S4). At the order level, the highest increase of relative abundance in AUD was observed for *Fusobacteriales* and the largest reduction for *Oscillospirales* (Supplementary Figure S5). Patients with AUD compared with HC presented with a reduced relative abundance of *Acutalibacteraceae* and several families containing short-chain fatty acid (SCFA) producing bacteria (*Ruminococcaceae*, *Oscillospiraceae*, *Butyricicoccaceae*, *Lachnospiraceae*, *Clostridiaceae*, and *Barnesiellaceae*, Figure 6). Patients with AUD were enriched in *Enterobacteriaceae*; however, confidence intervals were wide and crossed 0. Among the more abundant families in AUD patients, only *Burkholderiaceae*, *Fusobacteriaceae*, and *Xanthobacteraceae* had confidence intervals that did not cross 0 and were detected as significant in the random effects model. Changes at the family level were partially repeated at the genus level (Supplementary Figure S6), and several genera from *Lachnospiraceae* (*Scatomonas*, *Fusicatenibacter*, *Roseburia*, *Choladousia*, *Dorea_A*, *Lachnoclostridium*, *Roseburia*, *Eubacterium*), *Ruminococcaceae* (*Bittarella*), and *Butyricicoccaceae* (*Agathobaculum*) were decreased in AUD, and two genera from *Burkholderiaceae* (*Duodenibacillus*, *Parasutterella*) were increased.

### 2.4. Alcoholic Liver Disease versus Alcohol Use Disorder without Liver Disease

Patients with ALD showed a further reduction in *Firmicutes* and *Cyanobacteria*, and an increase of *Proteobacteria* compared to AUD without liver disease; other changes included a reduction of *Methanobacteriota* and a very slight increase in *Campylobacterota* (Supplementary Figure S7a). The F/B ratio did not differ significantly between ALD and AUD without liver disease (Supplementary Figure S10). Patients with ALD compared with AUD showed further enrichment of *Campylobacteriota*, an increase of *Gammaproteobacteria*, and a reduction of *Clostridia* and *Methanobacteria* (Supplementary Figure S7b). The most notable changes at the order level were a reduction of *Oscillospirales* and an increase of *Enterobacterales* in patients with alcoholic liver disease (Supplementary Figure S7c). ALD was associated with further reduction in *Acutalibacteraceae* and SCFA-producing families compared with AUD. Nine families were detected as more abundant in ALD, with the largest increase in *Enterobacteriaceae*, *Veillonellaceae*, *Neisseriaceae*, and *Campylobacteraceae* (Supplementary Figure S7d). Changes at the genus level included further reduction of genera from *Lachnospiraceae* (*Mediterraneibacter*, *Dorea*, *Scatomonas*, *Blautia_A*), *Ruminococcaceae* (*Ruminococcus_C*), and *Butyricicoccaceae* (*Agathobaculum*) families and an increase in several, potentially pathogenic genera such as *Streptococcus* in the ALD group (Supplementary Figure S7e).

### 2.5. Alcohol Liver Disease versus Other, Non-Alcoholic Causes of Liver Diseases

Compared with other causes of liver disease, ALD was characterized by lower relative abundances of *Verrucomicrobiota*, and a higher abundance of *Campylobacterota* and *Patescibacteria* (Supplementary Figure S8a). Interestingly, compared with other causes of liver disease, ALD was characterized by a lower abundance of *Clostridia*, an increase of *Bacilli*, and slight alterations of *Verrucomicrobiae*, *Campylobacteria*, and *Saccharimonadia* (Supplementary Figure S8b). At the order level, ALD was associated with the highest reduction of *Lachnospirales* and the highest increase in *Lactobacillales* (Supplementary Figure S8c). Compared to other causes of liver disease, ALD was characterized by a lower abundance of *Oscillospiraceae*, *Lachnospiraceae*, and *Butyricicoccaceae* (and multiple other families, Supplementary Figure S8d) and a higher abundance in 10 families, with *Lactobacillaceae* having the largest increase. Consistently, patients with ALD were enriched in the *Lactobacillus* genus compared with other causes of liver disease (Supplementary Figure S8e).

### 2.6. AUD and Liver Disease Cause Alterations in Predicted Functional Metagenomic Profiles

**Figure 7:**
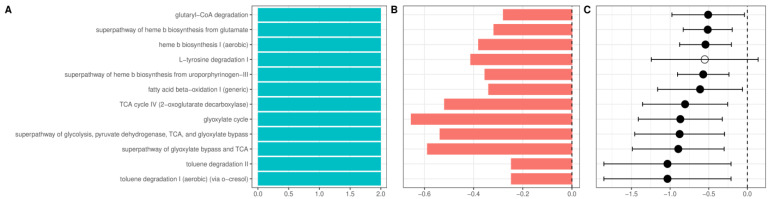
Changes in the relative abundance of the inferred metagenomic pathways between patients with alcohol use disorder and healthy controls (**A**). Number of datasets in which given pathway was detected as significant. (**B**). Change in the relative abundance of a given pathway in the combined dataset reported as log-ratio. Blue denotes bacteria more abundant in healthy controls, and red more abundant in alcohol use disorder. (**C**). Change in the relative abundance of a given pathway after summarizing results from individual datasets using the random-effects model, expressed as a mean difference in centered log ratios. Values larger than zero denote greater abundance in healthy controls. A full dot denotes a statistically significant effect. Error bars indicate 95% confidence intervals. Only pathways detected as significant in at least two datasets or significant in random-effects analysis are presented.

Multiple pathways enriched in AUD patients were related to heme synthesis. In addition, several aerobic pathways and pathways related to citrate (TCA) and glyoxylate cycle (Figure 7) were enriched in AUD. Patients with ALD showed further enrichment of pathways related to TCA and glyoxylate cycles and biosynthesis of heme. Additionally, among pathways with the largest increase in ALD was the LPS-related super pathway of (Kdo)2−lipid A biosynthesis; among other changes, the most consistent was the enrichment of multiple pathways related to menaquinol biosynthesis (Supplementary Figure S11). Recently, we have shown that alcoholic liver disease is associated with an increase in plasma concentration of a novel advanced glycation end product (AGE10). Synthetically obtained melibiose-derived AGE mimics this AGE10 epitope. We hypothesized that microbiome alterations could be associated with increased availability of melibiose in the gut. Melibiose can be delivered to the human organism from a plant diet or provided by gut microbiota. In dysbiosis, melibiose could be translocated to circulation thus contributing to AGE10 increase, whereas in healthy conditions, melibiose is hydrolyzed with microbial α-galactosidase. For this reason, we performed additional analysis of PICRUST2 data with the Kyoto Encyclopedia of Genes and Genomes as a reference database, targeting α-galactosidase (KEGG identifier K07407), which catalyzes the reaction of melibiose hydrolysis. The abundance of α-galactosidase did not differ significantly between healthy control, patients with AUD, and patients with ALD.

### 2.7. Diagnostic Accuracy of Deep Learning in Predicting the Disease Status

**Figure 8:**
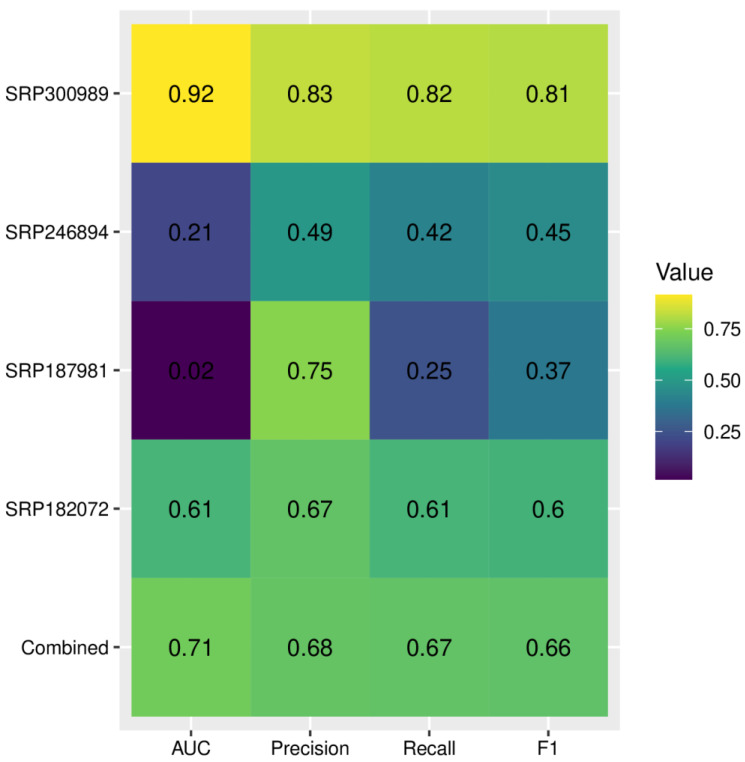
Accuracy of the deep learning model for microbiome-based differentiation between alcohol use disorder and healthy controls; AUC—area under the curve, F1—F1-score, harmonic mean of precision and recall. Larger values indicate better performance.

The metrics related to the diagnostic accuracy of deep learning are provided in Figure 8. Although the diagnostic accuracy for the combined dataset had an acceptable performance with 0.71 AUC, when applied to individual datasets we observed significant variance, with values of AUC ranging from 0.92 to only 0.02. The F1 score ranged from 0.37 to 0.81. The lowest AUC and F1-score were obtained for dataset SRP187981. To reduce overfitting, we have increased the lambda value of L2 regularization; however, it did not reduce variance across datasets and led to a further decrease of all metrics for the SRP187981 dataset. We hypothesized that weak performance on this dataset might be associated with significant class imbalance (i.e., a much larger number of AUD samples compared with HC). To combat this effect we performed random oversampling, which resulted in a significant increase of AUC to 0.25 (Supplementary Figure S12).

## 3. Discussion

The last decade has witnessed an exponential increase in studies that examine the microbiome in a wide range of diseases. During that time, several reports on microbiome alterations in AUD and ALD have been published [15,16,17,18,19,20]. The incorporation of multiple datasets in an analysis enables a more robust estimation of the dysbiosis patterns associated with AUD. However, differences in the methodologies used and the significant batch effect between studies make a direct comparison of the results unreliable. The use of raw reads from different datasets has allowed us to apply a consistent bioinformatical pipeline that reduced the variance associated with the choice of statistical tools. In addition, it allowed the estimation of the batch effect and then correcting for it. The batch effect in our study was a greater contributor to variance than the effect of disease status, a result consistent with previous reports that examined multiple microbial datasets [21]. The use of statistical methods that account for it resulted in finding consistent patterns of microbiome alterations associated with AUD and ALD.

Alpha-diversity analysis showed significant differences between patients with AUD, ALD, and other (nonalcoholic) liver diseases. No significant changes were detected between the AUD and HC groups. However, rarefication–extrapolation curves revealed that in most of the included studies, sequencing depth was not sufficient to capture the true diversity. Although the use of Hill’s numbers with asymptotic extrapolation provides more robust results than frequently used rarefication to common sequencing depth [22], it is still an estimation that could potentially differ from the true underlying diversity. For most of the datasets, beta diversity was significant, with the principal coordinate analysis (PCoA) plots showing some level of separation between the HC, AUD, and ALD groups.

Deep learning analysis has shown a strong variance in accuracy across datasets, even with increased L2-regularization (which is one of the most commonly used methods for the reduction of overfitting in neural networks). This shows that the good performance of deep learning models for microbiome-based predictions on individual datasets does not necessarily translate across datasets, confirming the common knowledge that training deep learning models requires diverse datasets. In the case of microbiome-based predictions, where the batch effect is often larger than the effect of disease status, this is especially crucial.

Our results show alterations in the microbiome that are consistent across multiple datasets. Patients with AUD were characterized by a reduction in *Firmicutes*, which was mostly attributed to a reduction in the *Clostridia* class. At the family level, this reduction can be explained by the lower abundance of *Ruminococcaceae*, *Lachnospiraceae*, *Oscillospiraceae*, and *Butyricicoccaceae* which contain multiple butyrate-producing bacteria. Patients with ALD showed a further reduction in most of these families compared to those with AUD without liver disease. The role of butyrate in maintaining gut health is multidirectional and is maintained through multiple mechanisms. Butyrate is the main source of energy for colonocytes [23]. Butyrate beta-oxidation induces physiological hypoxia in the colon [24]. Reduction of butyrate-producing bacteria, resulting in smaller availability of substrate for beta-oxidation leads to greater availability of oxygen, making the colonic environment more favorable to facultative anaerobes. This is reflected in our results: we have shown a significant increase in facultative anaerobes (such as the Enterobacteriaceae family) and a significant increase in multiple aerobic pathways in inferred metagenomic analysis. Due to the preferential use of butyrate as an energy source, a relatively small portion (about 5%) is absorbed into circulation [25]. However, even in small quantities, butyrate appears to have a strong anti-inflammatory effect on the host. This effect is achieved through multiple pathways, including interactions with G-protein coupled receptors (most notably GPR41 and GPR43 [26]), activation of nuclear factor kappaB (NF-kB), activation of PPAR-gamma, and inhibition of IFN-gamma signaling [27]. An additional mechanism through which the reduction of *Ruminococcaceae*, *Lachnospiraceae*, and other butyrate-producing bacteria could influence the course of AUD and its complications is through their effect on the intestinal barrier. Butyrate has been shown to have a protective effect on alcohol-induced intestinal barrier impairment, leading to a reduction in intestinal permeability [28]. The increase in intestinal permeability associated with the lower availability of butyrate allows increased endotoxin translocation, leading to sustained systemic inflammation [29]. Patients with AUD with and without liver disease have increased intestinal permeability; however, the increase is larger in patients with ALD [30]. This is consistent with our results, where patients with AUD had a smaller relative abundance, compared to HC, of the *Ruminococcaceae* family (which includes multiple intestinal barrier-protective bacteria), and patients with ALD showed further reduction of this family compared to AUD without liver disease. Patients with ALD are characterized by increased circulating levels of LPS, and the level of endotoxin correlates with the severity of liver injury [31]. Our results indicate that AUD is associated with a significantly higher abundance of *Proteobacteria* and that this increase is larger for patients with ALD. The increase in *Proteobacteria* is mainly attributed to the higher abundance of *Gammaproteobacteria*. The increase in this class is significantly correlated with higher serum levels of LPS [32]. Furthermore, the immunogenicity of LPS derived from *Proteobacteria* is significantly stronger compared to bacteria from other phyla [33]. Increased intestinal permeability combined with a greater abundance of highly immunogenic *Proteobacteria*-derived LPS are important contributors to systemic inflammation associated with AUD and ALD. At the family level, the most notable increase in bacteria from the *Proteobacteria* phylum was observed in *Enterobacteriaceae* and *Burkholderiaceae. Enterobacteriaceae* play an important role in the course of liver diseases. In patients with hepatic encephalopathy, and they have been shown to be associated with systemic inflammation and worsening of cognition [34]. Furthermore, *Enterobacteriaceae* are responsible for most cases of spontaneous bacterial peritonitis, which is the most common infection in patients with liver cirrhosis [35]. *Burkholderiaceae* further increases ethanol-associated inflammation. It is significantly correlated with IFN-gamma-inducible protein 10 (IP-10, sometimes called CXCL10), which exacerbated the inflammatory response in the murine model of ALD [36]. *Fusobacteria*, the phylum with the largest increase in AUD compared to HC in our study, further contributes to inflammation. It has been shown to be significantly correlated with higher levels of pro-inflammatory cytokines IL-2 and IL-13 [34]. In light of a report showing that supplementation with *Lactobacillus rhamnosus GG* ameliorates liver injury in a murine model [37], the increase in *Lactobacilli* in ALD compared to other causes of liver disease might appear paradoxical. However, a previous shotgun metagenomic study of ALD has shown that the increase in *Lactobacillus* was mostly attributed to oral species (such as *Lactobacillus salivarius*) and did not include *Lactobacillus rhamnosus*. We provide two potential mechanisms for the higher abundance of *Lactobacillus* in ALD compared to other causes of liver injury. First, it could be attributed to alcohol-induced disturbances in bile acids metabolism. Feces of patients with ALD had a lower concentration of deoxycholic acid (DCA) [38], which exhibits strong antimicrobial properties. Reduction in a DCA could result in a colonic environment more favorable for bacteria typically present in the oral microbiome (including *Lactobacillus*) [39]. Second, the higher abundance of *Lactobacillus* could be attributed to its metabolic abilities, namely, the ability to metabolize ethanol [40]. Inferred metagenomic analysis revealed some interesting alterations in AUD. The glyoxylate cycle enables the use of ethanol as a source of acetyl coenzyme A [41]. Enrichment of this pathway could be one of the adaptive strategies employed by bacteria more abundant in the AUD. Another interesting insight is provided by the enrichment of multiple pathways associated with the synthesis of heme. Dietary heme has been shown to alter the composition of the microbiome and increase intestinal inflammation [42]. Whether microbiome-derived heme has similar biological effects remains to be elucidated. 

Our study has an important limitation. Although we have provided a comprehensive examination of the microbiome changes associated with AUD and ALD, the exploratory nature of our study means that we cannot establish causality based on our findings. To fully understand the associations identified in our study, it is crucial to conduct further, mechanistic studies that aim to establish causality.

## 4. Materials and Methods

### 4.1. Characteristics of the Included Datasets and Overview of the Pipeline

We have included publicly available datasets from NCBI’s Sequence Read Archive (SRA) or European Nucleotide Archive (ENA), which provided raw 16S rRNA sequencing data and corresponding metadata for the following groups: (1) patients with AUD and healthy controls (HC), (2) patients with AUD without liver disease and patients with ALD, (3) patients with ALD and liver disease of other etiology. The characteristics of the included studies are presented in Table 1. The overview of the pipeline is illustrated in Supplementary Figure S1. Datasets providing samples for patients with AUD and HC were analyzed using two approaches: the combined dataset approach (where data from all qualifying studies was pooled for downstream quality control and statistical analysis) and the individual dataset approach (where each study was analyzed separately). Since only one dataset was available for both comparison of ALD with other causes of liver disease and with AUD (with respective accession IDs SRP185798 and SRP187981), they were analyzed using an individual study approach only. Statistical analysis was performed using R, version 4.1 [43].

### 4.2. Data Preparation

The entire workflow was run using Snakemake version 7.14.0 [44]. Primers were removed using Cutadapt [45] with a minimum read length set to 30 and other parameters set to default (i.e., a maximum error rate of 0.1). The reads were then merged (with a maximum number of mismatches set to 1 and minimum % identity of alignment set to 80), truncated to 250 bases, and quality filtered (with a maximum expected error of 1.0). Obtained sequences were denoised using the UNOISE3 algorithm implemented in USEARCH v11.0.667 [11,46], resulting in the generation of zero-radius operational taxonomic units (zOTUs). zOTUs are generally equivalent to OTUs (however, with some notable differences, e.g., in contrast to normal OTUs, zOTUs undergo denoising) with a 100% identity threshold, that is, each zOTU represents one correct biological sequence. The use of a 100% identity threshold is the optimal approach for the data from the V4 hypervariable region sequencing [47]. Taxonomy was determined using DECIPHER IDTAXA [12], with GTDB version 07-RS207 as a reference database [48]. The obtained zOTU table, corresponding metadata, and taxonomy have been imported into the Phyloseq object for downstream analysis [49]. Functional abundance prediction was performed using PICRUSt 2 [50] with the Metacyc database as a reference. The plots were generated using ggplot2 version 3.3.6 [51].

### 4.3. Variance Contribution and Beta-Diversity

Before calculating variance contribution and beta diversity, the zOTU tables were normalized using PhilR [52]. The beta diversity was then assessed using Euclidean distance. The use of PhilR with Euclidean distance has several advantages over commonly used methods for asserting beta diversity. It incorporates phylogenetic information, but contrary to other phylogenetically-aware methods (such as UniFrac), it accounts for the compositional nature of microbiome datasets, which is crucial for the reduction of spurious results [53]. The statistical significance of beta diversity was determined using PERMANOVA with 999 permutations. The variance contribution was calculated using redundancy analysis (RDA, implemented in the Vegan package version 2.6–2 [54]). The disease status (both for the individual and combined dataset approach) and the SRA identifier (only for the combined approach) were used as constraining variables.

### 4.4. Alpha Diversity

To account for differences in the read depth between samples (which influences alpha-diversity estimates [55]), samples were normalized using asymptotic extrapolation. In contrast to ordinary rarefication to a given read length, the asymptotic extrapolation does not require throwing away valid reads and, as a result, enables a more accurate estimation of alpha diversity [22]. Alpha diversity was measured using Hill numbers with q values equal to 0 (species richness), 1 (Hill–Shannon diversity), and 2 (Hill–Simpson diversity). The use of Hill numbers solves several problems associated with traditional alpha diversity measures such as Shannon or Simpson diversity; it offers conceptually simpler interpretation (e.g., reduction of 1/3 of the species in a community results in a 1/3 reduction of Hill diversity; in contrast for both ordinary Shannon and Simpson indices, the reduction is much smaller than anticipated). For a more comprehensive description of asymptotic estimation and Hill numbers, we refer the reader to the seminal paper by Chao et al. [22]. Both asymptotic estimation and Hill number calculation were performed using the iNEXT package [56].

### 4.5. Differential Abundance Testing

Differential abundance testing was performed using ANCOM-BC [13]. ANCOM-BC is one of the compositionally aware methods for DA testing which accounts for uneven sampling using a novel method of bias correction. It has been shown to significantly reduce FDR compared to other approaches while maintaining adequate statistical power [13,57]. A comprehensive discussion of the statistical properties and assumptions underlying ANCOM-BC can be found in the manuscript by Lin et al. [13]. Differential abundance testing was performed at phylum, class, order, family, and genus levels. Since sequencing of individual subregions of 16S rRNA (e.g., V4) does not achieve the taxonomic resolution required for accurate classification of species [58], differential abundance testing at this level was not performed.

### 4.6. Combined Dataset Analysis

In the combined approach, all datasets that provided samples for patients with AUD and HC were analyzed as one dataset, with a pipeline analogous to the individual study approach. To obtain globally aligned reads (i.e., starting and ending at the same position of 16S rRNA), we have used 515F (5′-GTGYCAGCMGCCGCGGTAA-3′) and 805R (5′-GACTACHVGGGTATCTAATCC-3′) primers. The dataset DRP003174 was excluded from the combined approach due to sequencing of the V1–V2 region, which could not be globally aligned with V3–V4. The significance testing was performed analogously to the individual study approach (with one notable difference of using SRA ID as a concomitant variable in ANCOM-BC). Alpha-diversity and CLR-transformed microbial abundances from the individual study approach were transformed to Agresti’s generalized odds ratios using the genodds package [59] and summarized with the random-effects model using the meta package [60].

### 4.7. Deep Learning Analysis

We used the PopPhy-CNN convolutional neural network for taxonomy-based prediction of disease status (AUD versus HC) [61]. The model was trained using default settings (L2Lambda = 0.001). Due to the high variance across datasets, we re-trained the model with increased lambda values (0.003 and 0.01) to strengthen the L2-regularization. L2-regularization reduces the weights features by adding the sum of squares of feature weights to the loss function. Higher values of lambda result in bigger punishment of large feature weights. Due to a significant class imbalance in one of the datasets, we performed random oversampling (ROS). ROS is a technique that combats class imbalance by multiplying randomly chosen samples from a minority class in the training set. The metrics used for the evaluation of the model were precision, recall, an area under the curve (AUC), and F1-score (harmonic mean of precision and recall).

## 5. Conclusions

In conclusion, we have shown that the batch effect is one of the largest contributors to variance across different datasets that examine AUD. The most consistent changes in the microbiome in AUD were related to a reduction in SCFA-producing bacteria and an increase in bacteria associated with inflammation. Inferred metagenomic analysis showed that ALD is associated with an increase in multiple pathways related to bacterial heme synthesis, which so far has not been studied in the context of alcoholic liver disease. Deep learning analysis has shown significant variance in the microbiome-based prediction of AUD. Our findings confirm that AUD is associated with negative microbiome alterations, which could be mechanistically linked to liver injury.

## Figures and Tables

**Table 1:** Characteristics of the included datasets.

Dataset	Region	Platform	Primers	Country	Samples
DRP003174 [15]	V1–V2	454 GS FLX Titatnium and Junior (Roche Applied Science, Penzberg, Upper Bavaria, Germany)	27F/338R	Japan	AUD *n* = 16, HC *n* = 40
SRP182072 [16]	V3–V4	Illumina HiSeq (Illumina, USA)	342F/806R	Norway	AUD *n* = 21, HC *n* = 30
SRP185798 [17]	V3–V4	Illumina MiSeq and HiSeq (Illumina, USA)	341F/805R	USA	ALD *n* = 6, NALD *n* = 54
SRP187981 [18]	V4	Illumina MiSeq (Illumina, USA)	515F/806R	USA, Mexico, Europe	ALD *n* = 31, AUD *n* = 30, HC *n* = 4
SRP246894 [19]	V4	Illumina MiSeq (Illumina, USA)	515F/806R	USA	AUD *n* = 55, HC *n* = 159
SRP300989 [20]	V3–V4	Illumina MiSeq (Illumina, USA)	338F/806R	China	ALD *n* = 38, HC *n* = 27

## Data Availability

The raw sequences used in this study are deposited in the SRA with the following accession numbers: DRP003174, SRP182072, SRP185798, SRP187981, SRP246894, and SRP300989.

## Supplementary Materials

The following supporting information can be downloaded at: https://www.mdpi.com/article/10.3390/ijms24032461/s1.
