# Supplementary material for: Microbiome Alterations in Alcohol Use Disorder and Alcoholic Liver Disease
Source: Int J Mol Sci. 2023 Jan 27;24(3):2461. doi: 10.3390/ijms24032461 (PMC9916746; doi:10.3390/ijms24032461)
Supplement: Supplementary file 1 [file ijms-24-02461-s001.zip › ijms-2078223-supplementary.pdf]

# Supplementary file

# Title: Microbiome alterations in alcohol use disorder and alcoholic liver disease

Kamil Litwinowicz[1]*, Andrzej Gamian[2]

[1] Department of Biochemistry and Immunochemistry, Wroclaw Medical University

[2] Hirszfeld Institute of Immunology and Experimental Therapy, Polish Academy of Sciences

* Correspondence: kamil.litwinowicz@student.umw.edu.pl

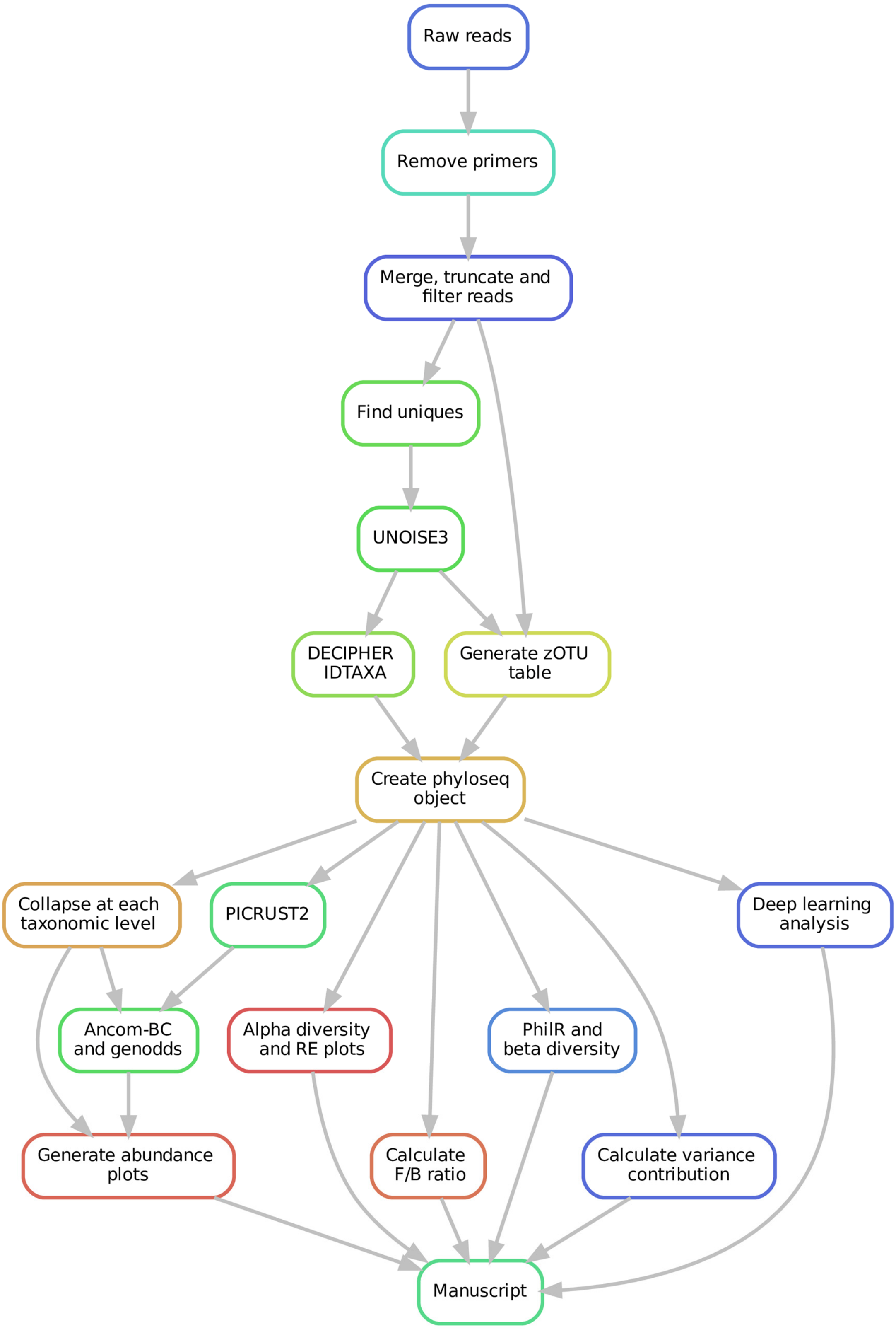


*Supplementary Figure S1. Simplified overview of the analytical pipeline*

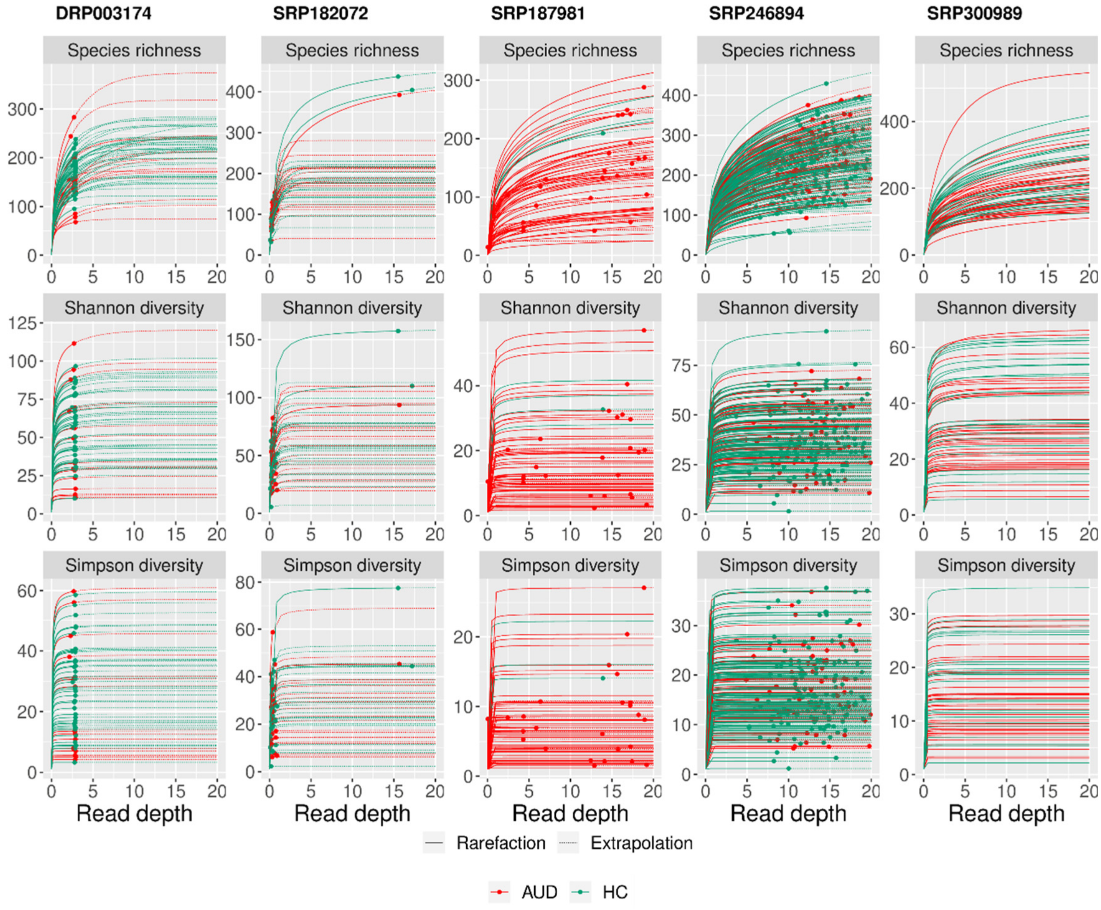


*Supplementary Figure S2. Rarefication-extrapolation curves for alpha-diversity analyses*

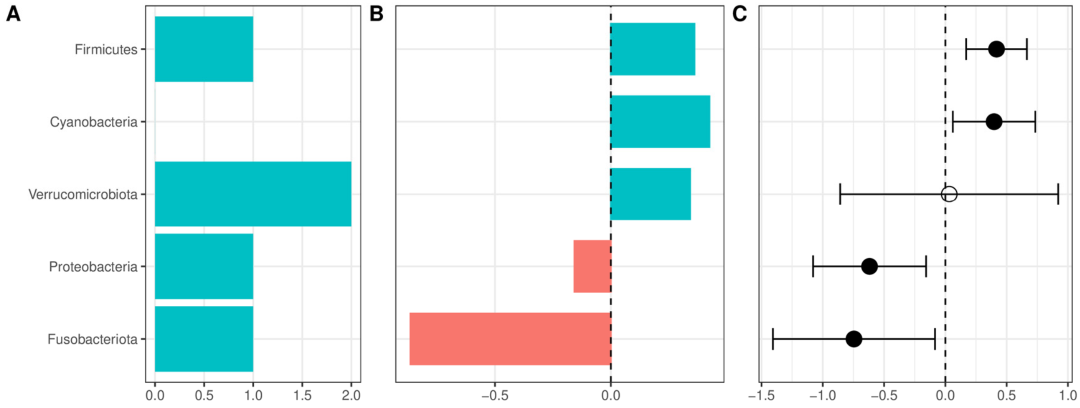


*Supplementary Figure S3. Changes in the relative abundance between patients with alcohol use disorder and healthy controls at the phylum level. a. Number of datasets in which the given phylum was detected as significant. b) Change in the relative abundance of a given phylum in the combined dataset reported as log-ratio. Blue denotes bacteria more abundant in healthy controls, and red more abundant in alcohol use disorder. c. Change in the relative abundance of a given phylum after summarizing results from individual datasets using the random-effects model, expressed as a mean difference in centered log ratios. Values larger than zero denote*

*greater abundance in healthy controls. A full dot denotes a statistically significant effect. Error bars indicate 95% confidence intervals. Only bacteria detected as significant in at least two datasets or significant in random-effects analysis are presented.*

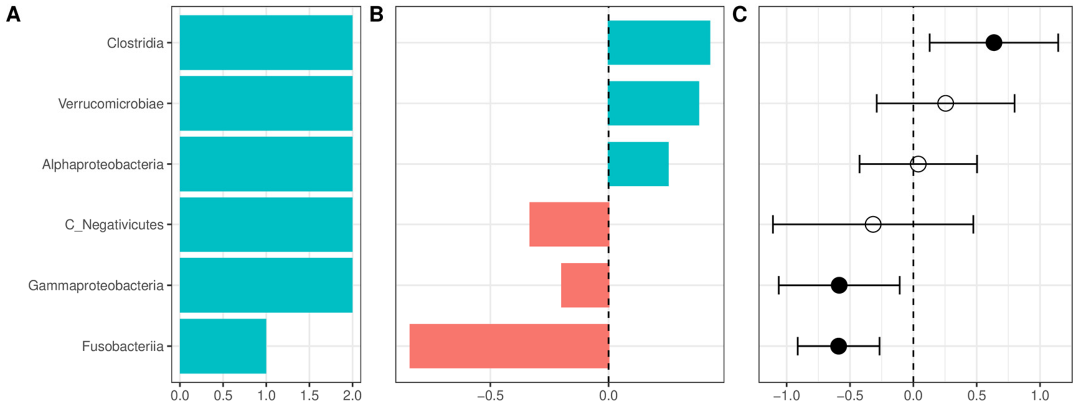


*Supplementary Figure S4. Changes in the relative abundance between patients with alcohol use disorder and healthy controls at the class level. a. Number of datasets in which the given class was detected as significant. b) Change in the relative abundance of a given class in the combined dataset reported as log-ratio. Blue denotes bacteria more abundant in healthy controls, and red more abundant in alcohol use disorder. c. Change in the relative abundance of a given class after summarizing results from individual datasets using the random-effects model, expressed as a mean difference in centered log ratios. Values larger than zero denote greater abundance in healthy controls. A full dot denotes a statistically significant effect. Error bars indicate 95% confidence intervals. Only bacteria detected as significant in at least two datasets or significant in random-effects analysis are presented.*

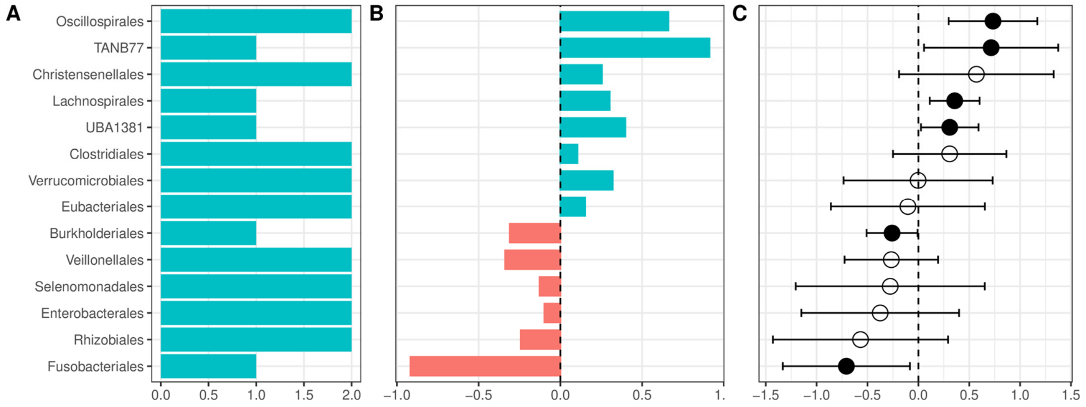


*Supplementary Figure S5. Changes in the relative abundance between patients with alcohol use disorder and healthy controls at the order level. a. Number of datasets in which the given order was detected as significant. b) Change in the relative abundance of a given order in the combined dataset reported as log-ratio. Blue denotes bacteria more abundant in healthy controls, and red more abundant in alcohol use disorder. c. Change in the relative abundance of a given order after summarizing results from individual datasets using the random-effects model, expressed as a mean difference in centered log ratios. Values larger than zero denote greater abundance in healthy controls. A full dot denotes a statistically significant effect. Error*

*bars indicate 95% confidence intervals. Only bacteria detected as significant in at least two datasets or significant in random-effects analysis are presented.*

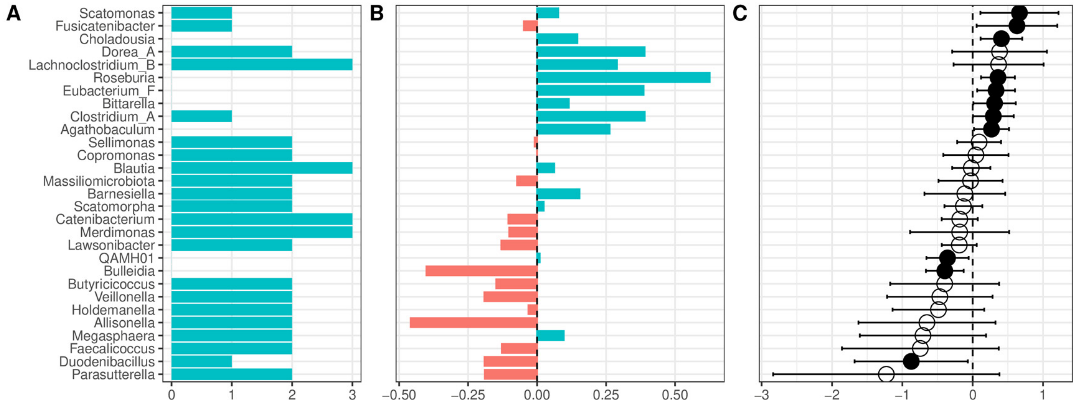


*Supplementary Figure S6. Changes in the relative abundance between patients with alcohol use disorder and healthy controls at the genus level. a. Number of datasets in which the given genus was detected as significant. b) Change in the relative abundance of a given genus in the combined dataset reported as log-ratio. Blue denotes bacteria more abundant in healthy controls, and red more abundant in alcohol use disorder. c. Change in the relative abundance of a given genus after summarizing results from individual datasets using the random-effects model, expressed as a mean difference in centered log ratios. Values larger than zero denote greater abundance in healthy controls. A full dot denotes a statistically significant effect. Error bars indicate 95% confidence intervals. Only bacteria detected as significant in at least two datasets or significant in random-effects analysis are presented.*

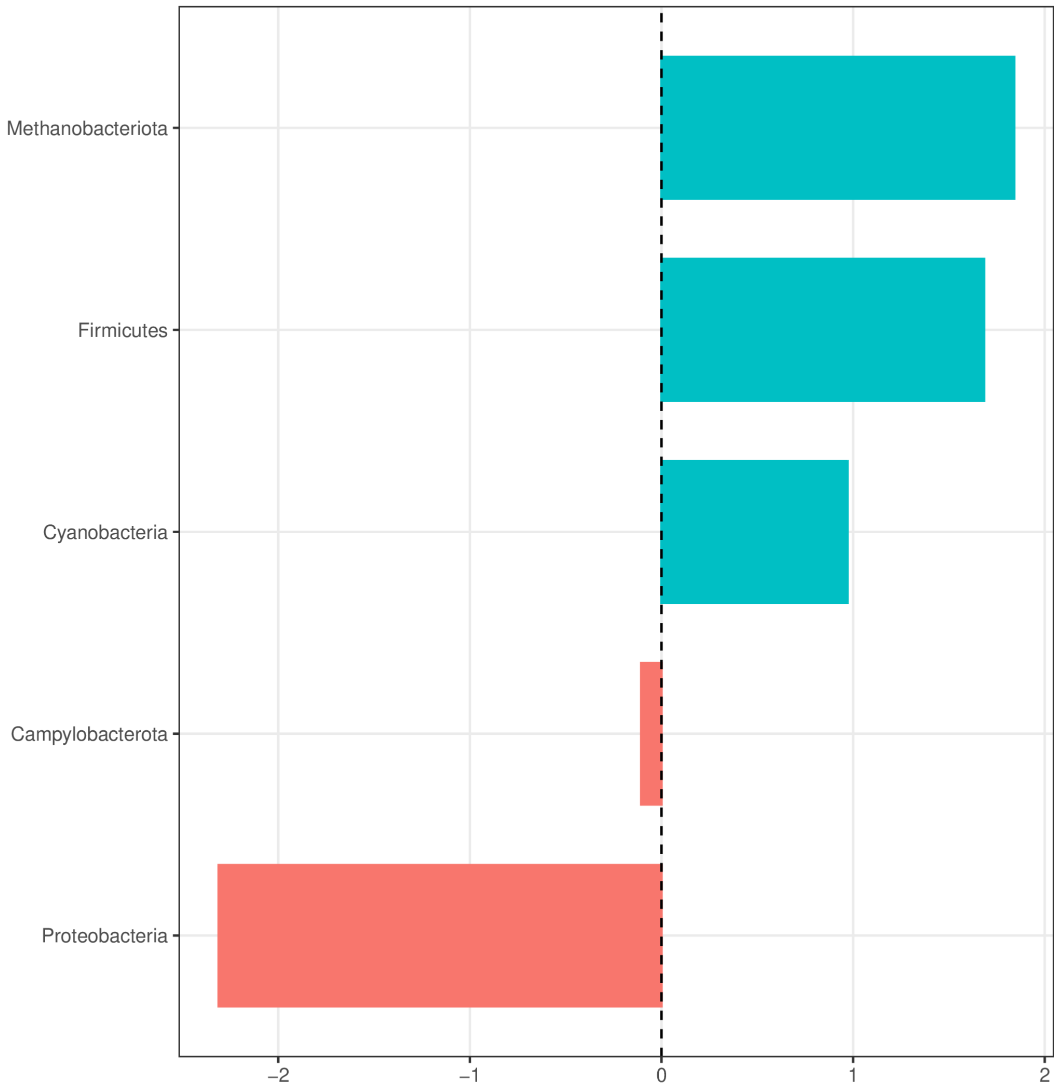


*Supplementary Figure S7. Changes in the relative abundance of bacteria at different taxonomy levels between patients with alcoholic liver disease and alcohol use disorder, expressed as log-ratios. Only bacteria detected as significant in ANCOM-BC are presented. Values greater than 0 indicate larger abundance in alcohol use disorder without liver disease. a) Phylum*

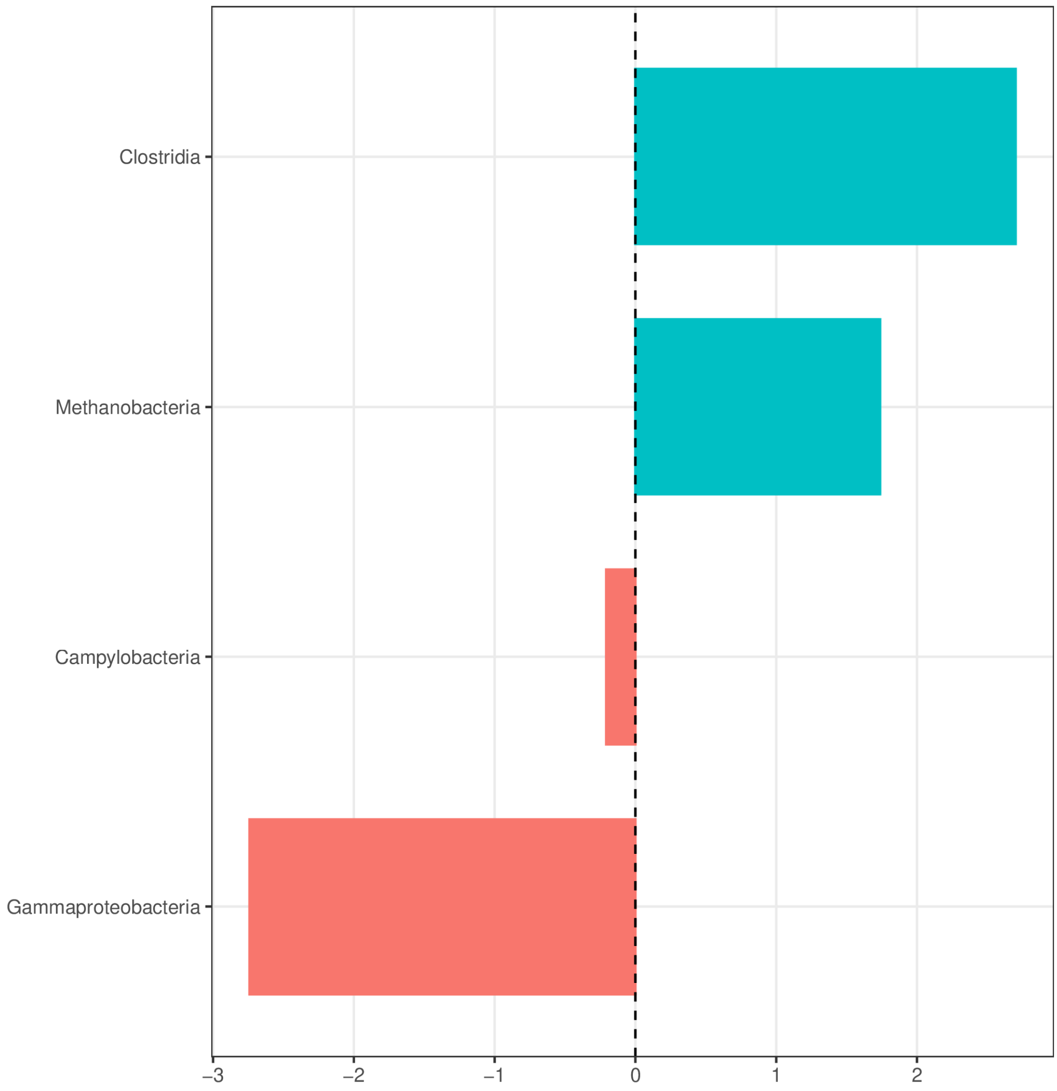


*Supplementary Figure S7. b) Class*

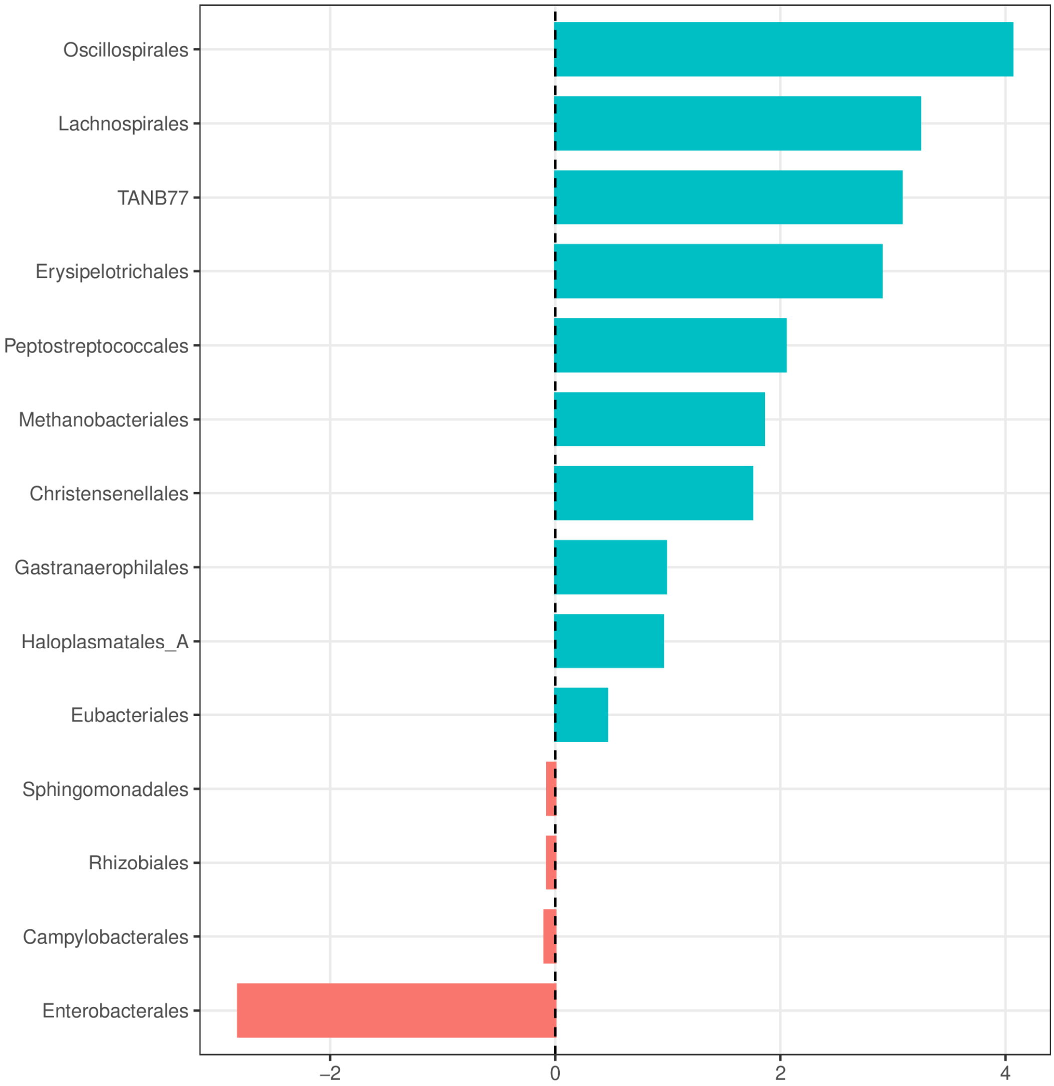


*Supplementary Figure S7. c) Order*

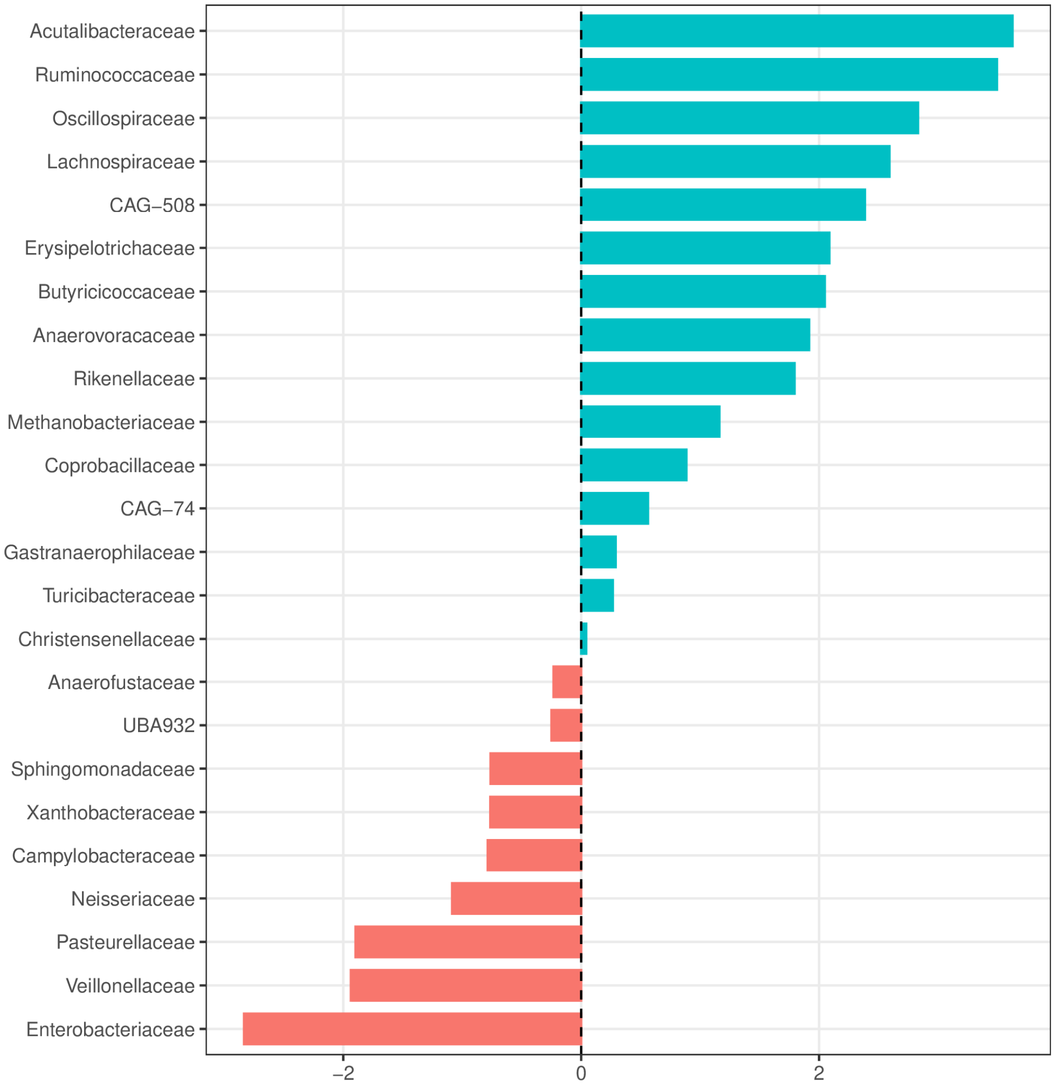


*Supplementary Figure S7. d) Family*

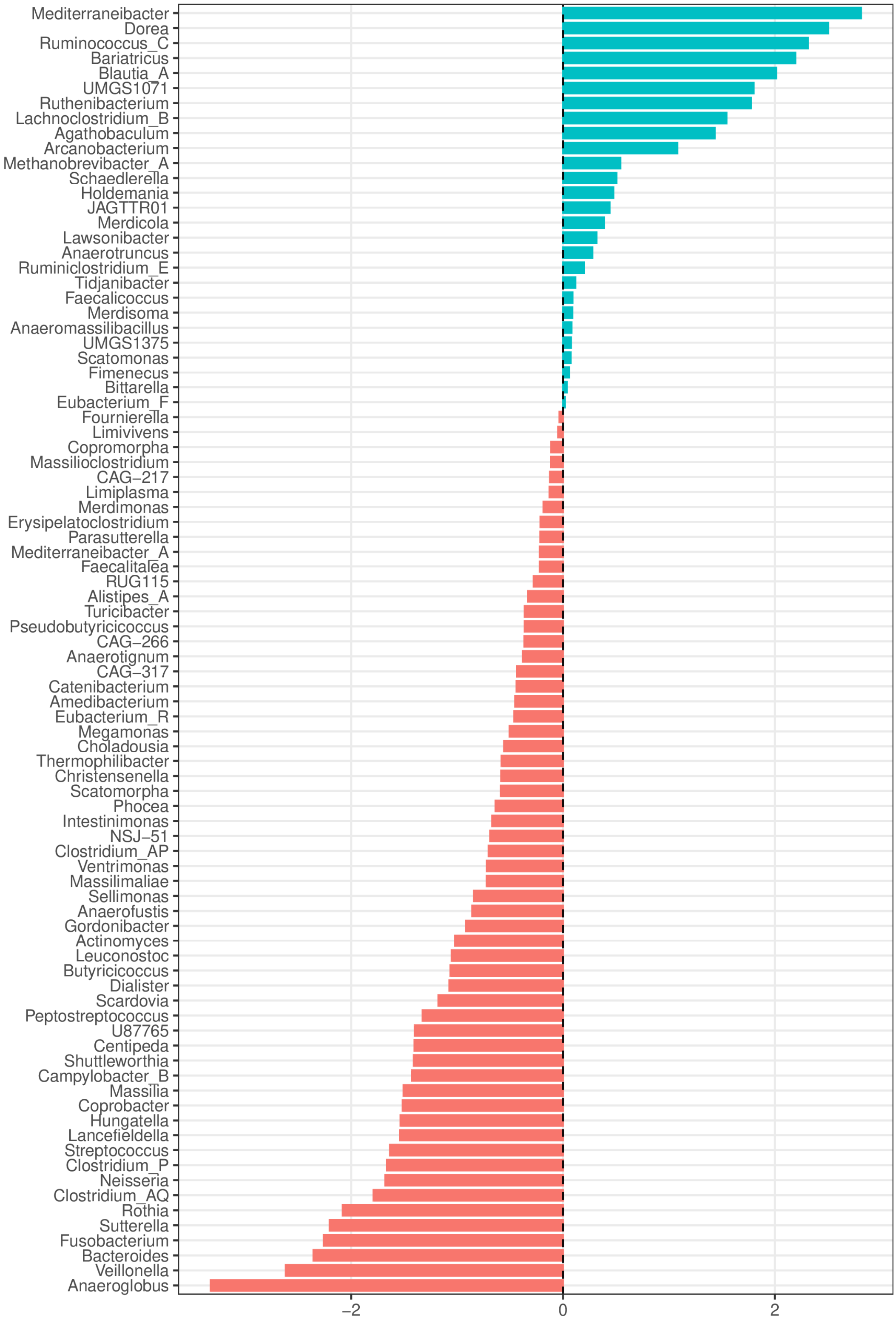


*Supplementary Figure S7. e) Genus*

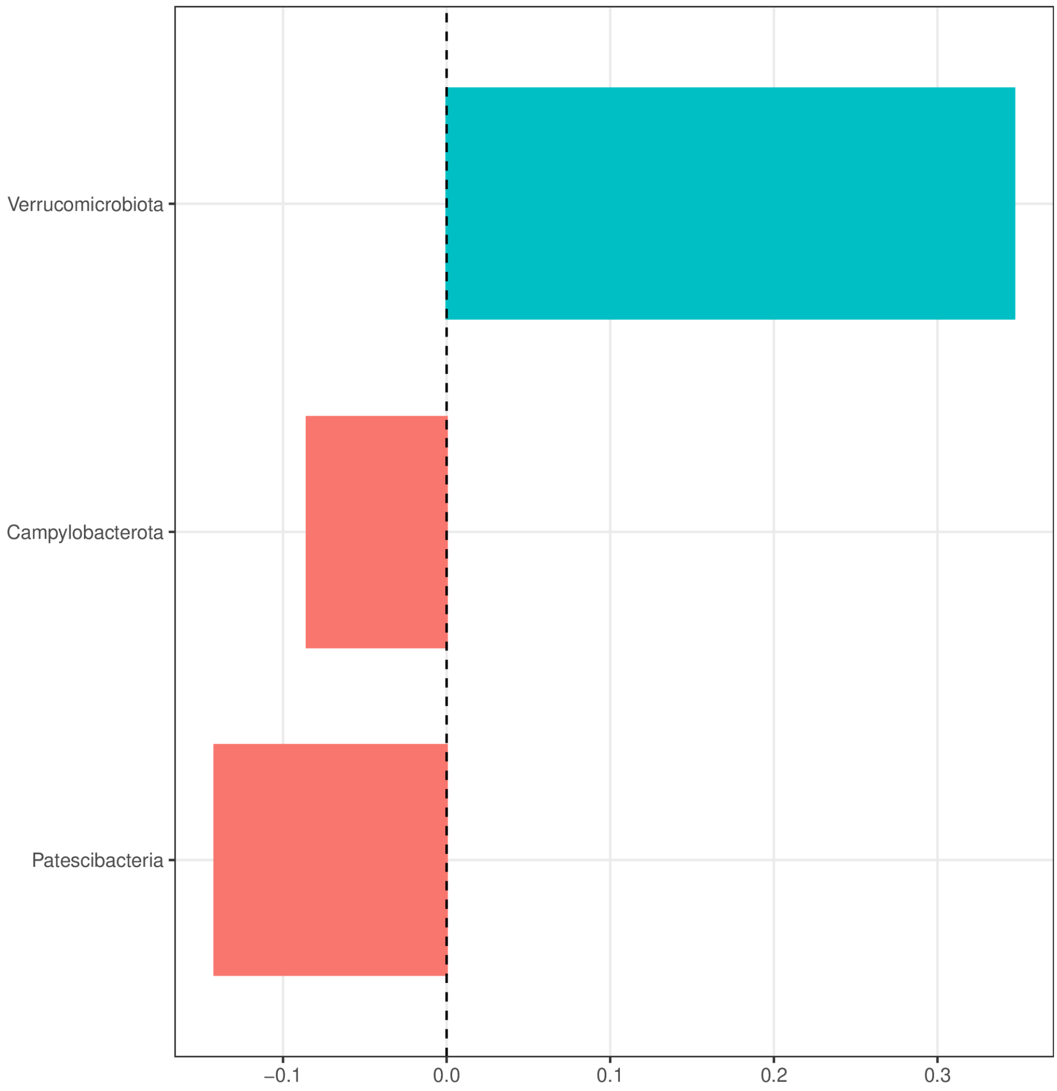


*Supplementary Figure S8. Changes in the relative abundance of bacteria at different taxonomy levels between patients with alcoholic liver disease and other causes of liver disease, expressed as log-ratios. Only bacteria detected as significant in ANCOM-BC are presented. Values greater than 0 indicate larger abundance in alcohol use disorder without liver disease. a) Phylum*

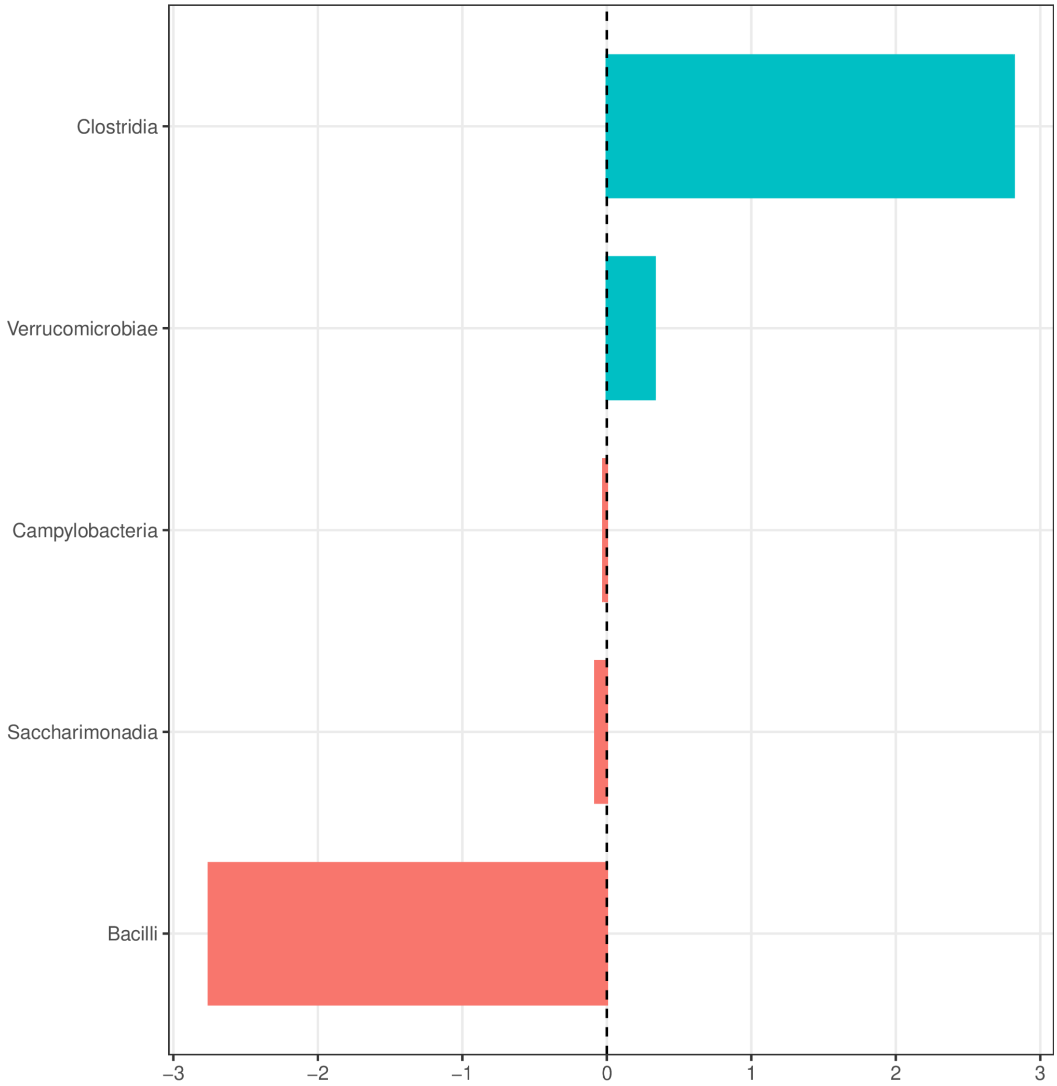


*Supplementary Figure S8. b) Class*

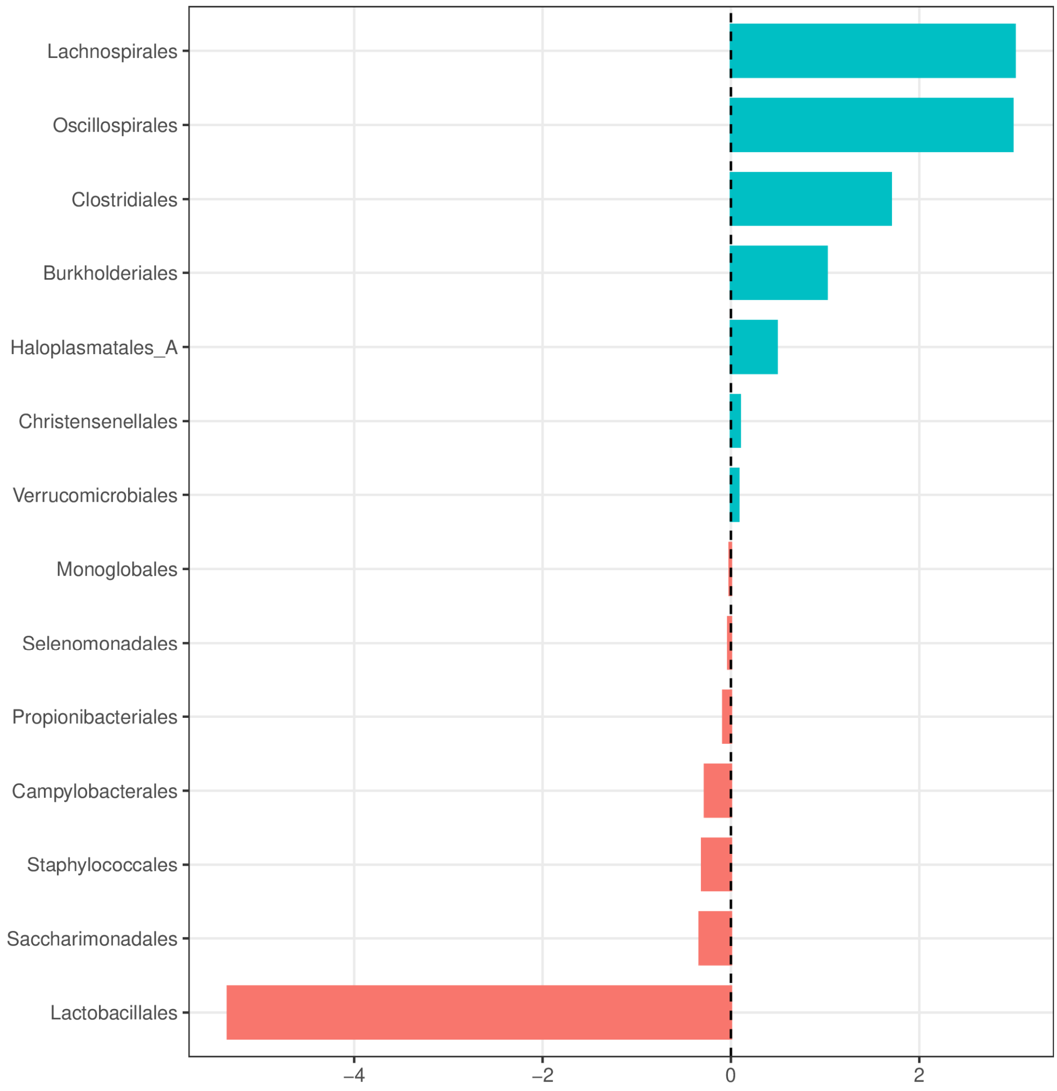


*Supplementary Figure S8. c) Order*

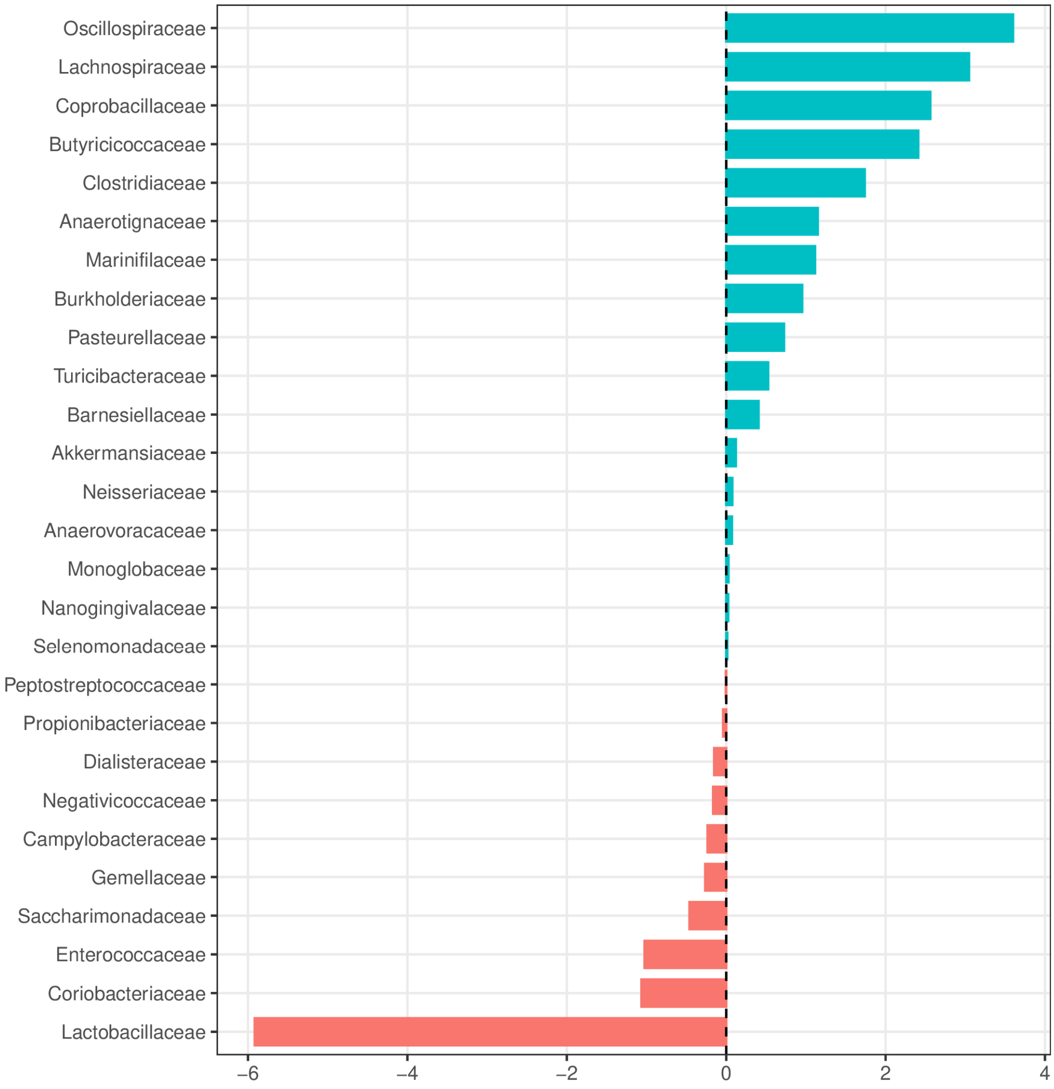


*Supplementary Figure S8. d) Family*

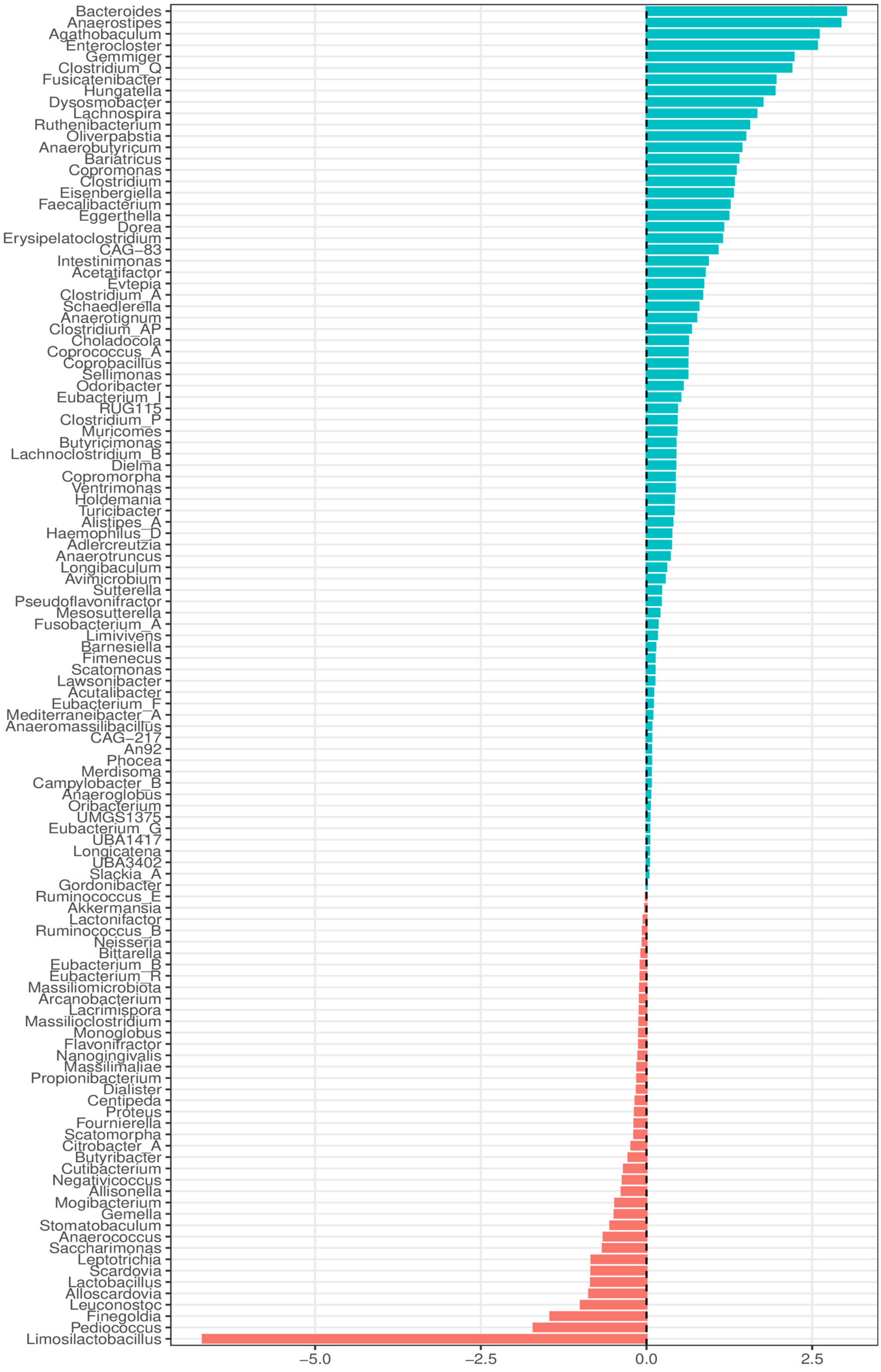


*Supplementary Figure S8. e) Genus*

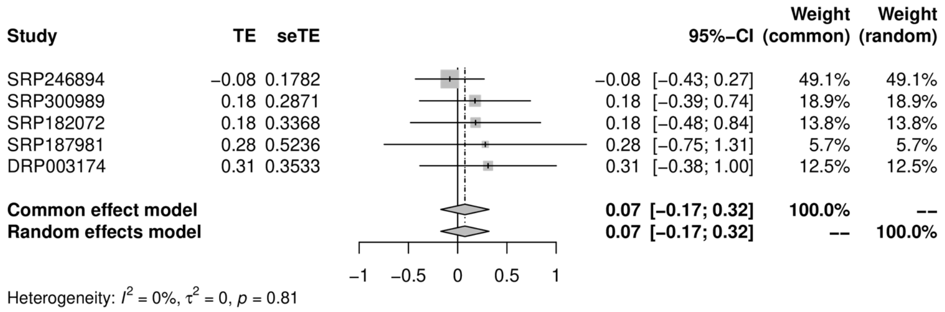


*Supplementary Figure S9. Comparison of Firmicutes/Bacteroidetes (F/B) ratio between patients with alcohol use disorder and healthy control. Values larger than 0 denote greater F/B ratio in healthy controls.*

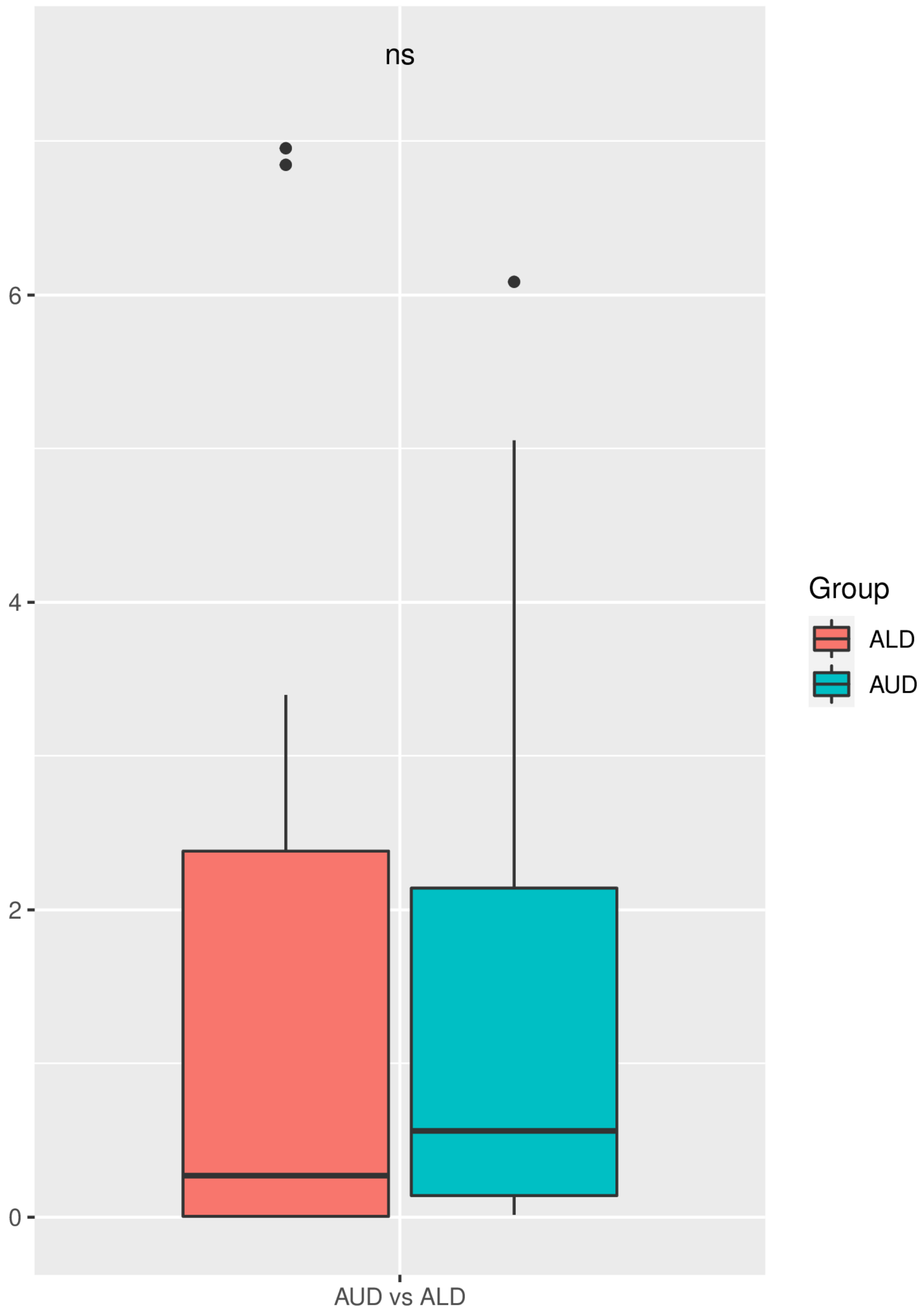


*Supplementary Figure S10. Comparison of Firmicutes/Bacteroidetes (F/B) ratio between AUD and ALD; AUD - alcohol use disorder, ALD - alcoholic liver disease, ns - non-significant result*

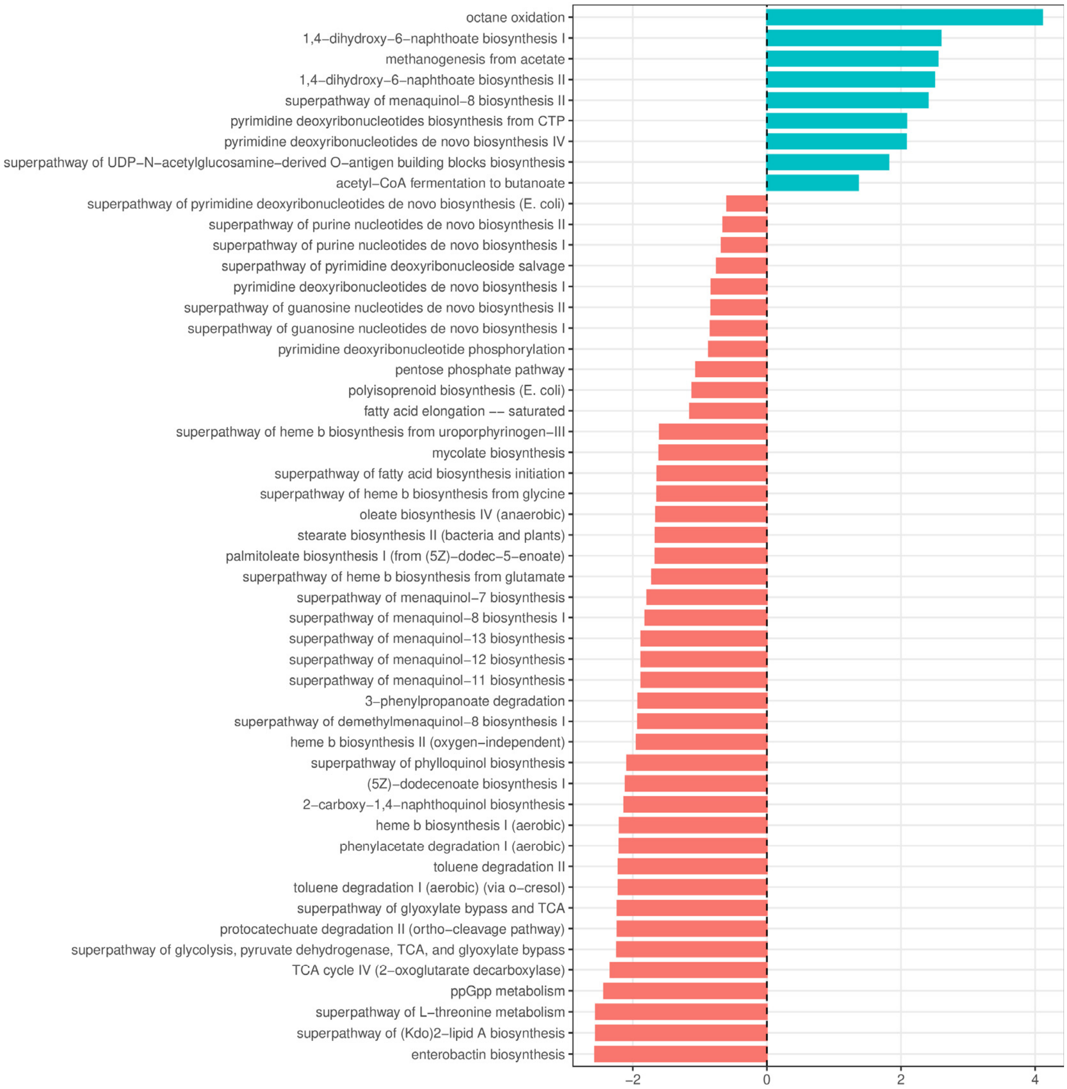


*Supplementary Figure S11. Changes in the relative abundance of the inferred metagenomic pathways between patients with alcoholic liver disease and alcohol use disorder, expressed as log-ratios. Only pathways detected as significant in ANCOM-BC are presented. Values greater than 0 indicate larger abundance in alcohol use disorder without liver disease.*

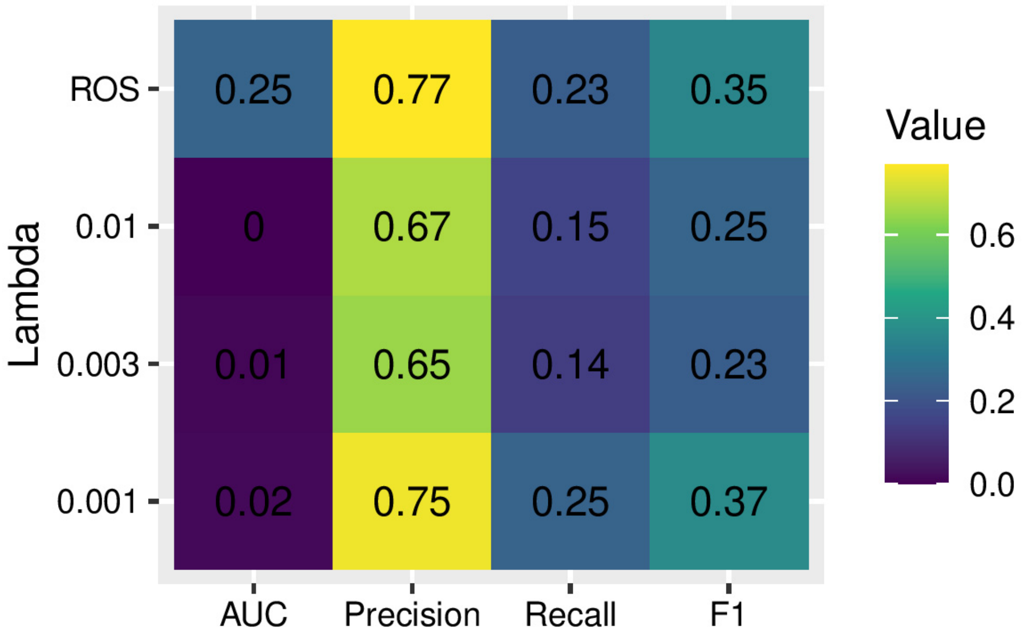


*Supplementary Figure S12. Accuracy of deep learning for prediction of disease status for the SRP187981 dataset with different values of lambda for L2 regularization and with inclusion of random oversampling (ROS)*
